# Influence of micropatterned substrates on keratocyte phenotype

**DOI:** 10.1038/s41598-020-62640-5

**Published:** 2020-04-21

**Authors:** Promita Bhattacharjee, Brenton L. Cavanagh, Mark Ahearne

**Affiliations:** 1 Trinity Centre for Biomedical Engineering, Trinity Biomedical Sciences Institute, Trinity College Dublin, University of Dublin, Dublin, Ireland; 2 Department of Mechanical and Manufacturing Engineering, School of Engineering, Trinity College Dublin, University of Dublin, Dublin, Ireland; 3 Cellular and Molecular Imaging Core, Royal College of Surgeons in Ireland, Dublin, Ireland

**Keywords:** Biomaterials - cells, Biomedical engineering, Tissue engineering

## Abstract

Substrate topographic patterning is a powerful tool that can be used to manipulate cell shape and orientation. To gain a better understanding of the relationship between surface topography and keratocyte behavior, surface patterns consisting of linear aligned or orthogonally aligned microchannels were used. Photolithography and polymer molding techniques were used to fabricate micropatterns on the surface of polydimethylsiloxane (PDMS). Cells on linear aligned substrates were elongated and aligned in the channel direction, while cells on orthogonal substrates had a more spread morphology. Both linear and orthogonal topographies induced chromatin condensation and resulted in higher expressions of keratocyte specific genes and sulfated glycosaminoglycans (sGAG), compared with non-patterned substrates. However, despite differences in cell morphology and focal adhesions, many genes associated with a native keratocyte phenotype, such as keratocan and ALDH3A1, remain unchanged on the different patterned substrates. This information could be used to optimize substrates for keratocyte culture and to develop scaffolds for corneal regeneration.

## Introduction

Located at the front of the eye, the cornea is the transparent outer component of the eye, responsible for focusing light into the eye and protecting the internal structure from external irritations^1^. The cornea comprises of five main layers: epithelium, Bowman’s layer, stroma, Descemet’s membrane and endothelium^2,3^. The stroma is the major structural and functional unit of cornea constituting 90% of the total corneal thickness. The collagen fibril structure in the stroma allows it to be transparent which is vital for preserving vision^4^.

Aligned collagen nanofibrils in stromal extracellular matrix (ECM) (mainly collagen I and V^5–8^) form layers arranged orthogonally to one another. Keratocytes, the major cellular component of stroma, are scattered within these layers. Corneal transparency is attributed to the highly ordered orthogonal distribution of fibril layers, spacing between collagen fibrils, modulation of the diameter of collagen fibril attained by proteoglycans (i.e. keratocan and lumican^9–12^) and crystallin proteins located within the cells. Keratocytes are dendritic in nature with expanded cellular network and compact cell body, enabling them to construct a three-dimensional network of interconnected cells^13,14^. Upon injury the cells lose their dendritic shape and change phenotype becoming fibroblastic or myofibroblastic.

Globally around 10 million suffer from vision impairment resulting from corneal injury^13^. Annually around 30,000–40,000 corneal graft replacements are performed within USA and Europe. While rejection rates are low (under 10%)^13^, for high-risk patients the rejection rate can increase to approximately 49% and continues to increase over the patient’s life. The limited availability of transplantable donated corneas exacerbates this problem. Synthetic keratoprostheses have been used as an alternative of allografts, however there is a relatively high rejection rate associated with these implants^13^. Biomimetic approaches have also been undertaken to fabricate bioengineered corneas that mimics the structure and function of human corneal extracellular matrix using a variety of natural and synthetic biomaterials^15–20^. One of the challenges with this approach is how to support transplanted keratocytes to develop well-ordered, aligned extracellular matrix.

An additional challenge is that keratocytes are quiescent *in situ*. To gain a sufficient number of cells for transplantation, if is necessary to expand keratocytes isolated for donor tissue in serum-supplemented media. This results in the cells becoming fibroblastic and alters their morphology and ECM deposition. Upon the removal of serum, a partial return to a native keratocyte phenotype is achievable^21–23^. In this study, we wanted to investigate if topographical cues could influence the restoration of a keratocyte phenotype in serum free media after the cells were initially expanded on culture plastic in serum-supplemented media.

Several studies have reported that phenotype and morphology of keratocytes are notably influenced by surface geometry and topography such as parallel linear grooves^24–27^ or concentric circles^13^. In many of these studies, keratocytes stretch and aligned in the groove direction. Despite the cells morphology not retaining a dendritic, *in-vivo* like morphology, there was an increase in keratocyte phenotype markers resulting from the presence of the grooves. The relationship between changes in keratocyte phenotype and morphology *in-vitro* that result from topographical cues has not previously been examined in detail and required further analysis. To gain a better understanding of the keratocytes morphology-phenotype relationship three different substrate topographies were used, one consisting of linear aligned channels (termed line) and two with orthogonally aligned channels with different dimensions (termed lattice and micropit). Unpatterned PDMS and standard tissue culture plate (TCP) were used as controls. Cell shape, orientation, chromatin condensation, focal adhesion area and length, migration speed and direction, stiffness, sulfated glycosaminoglycan (sGAG) release and gene expression were all examined.

## Results

### Characterization of substrate topography

**Figure 1:**
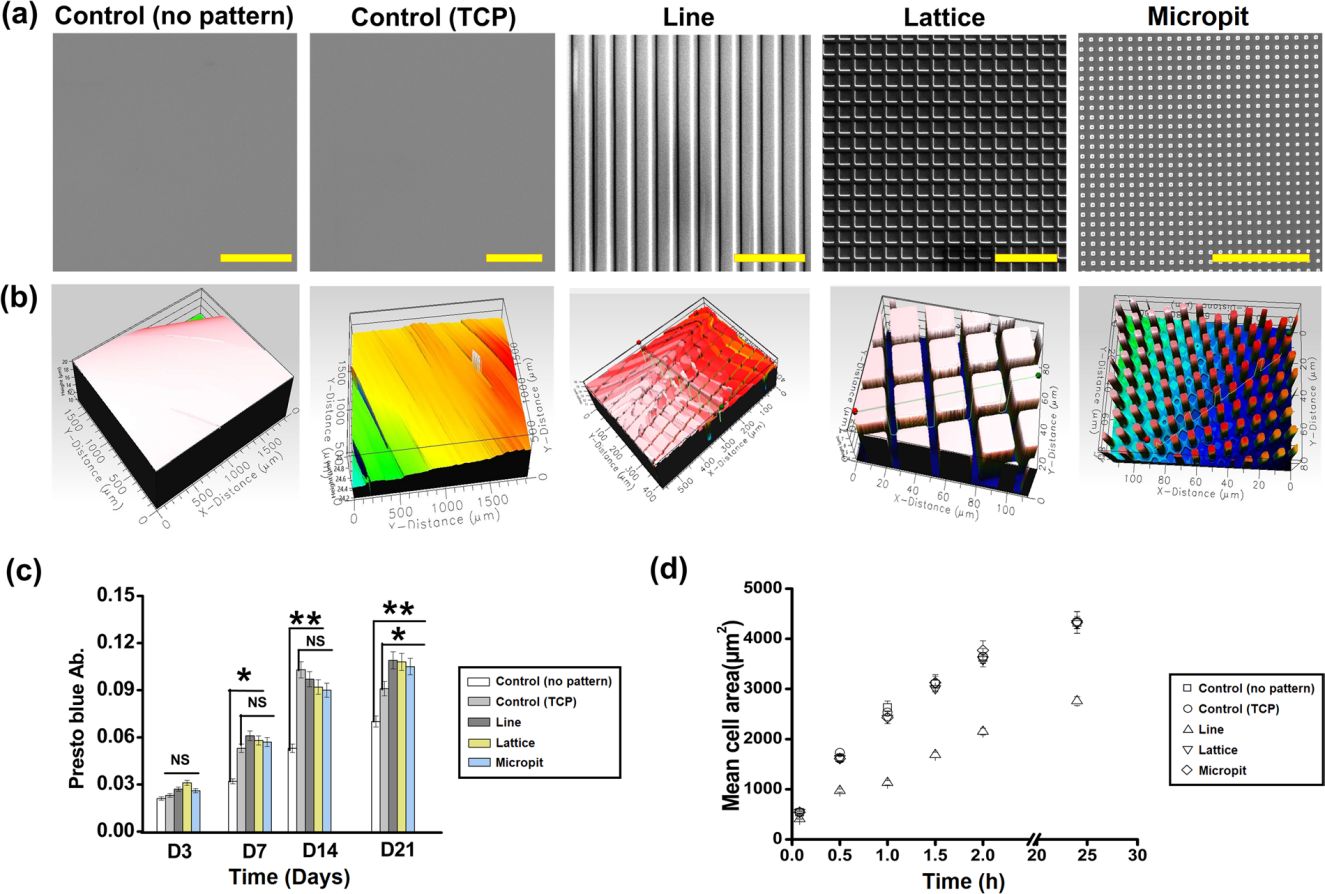
Substrates with micro patterned surfaces for cell culturing. (**a**) Scanning electron micrographs (scalebar = 100 µm) and (**b**) white light image of different PDMS and TCP substrates with or without line, lattice and micropit topographies; (**c**) growth kinetics of keratocytes seeded on substrates over 21 days; (**d**) fluorescent micrograph quantification was used to calculate mean cell area. Data are presented as mean ± SD, n = 20 (**p < 0.01; *p < 0.05).

Substrates were fabricated that consisted of linear aligned microchannels (line) or orthogonally aligned microchannels (lattice and micropit). Substrates without any microchannels were used as a control. To examine the respective micropatterns, scanning electron microscopy (SEM) and white light interferometry were used (Fig. 1(a,b)). SEM images show that the patterns transferred to the PDMS successfully without any noticeable defects. White light interferometry was used to quantify the line depth of the patterns (15 min plasma etching, ~3.5 ± 0.5 µm) and the average surface roughness (RMS ~ 275–305 nm).

### Cell proliferation and size

The cell growth kinetics were evaluated using a presto blue assay over 21 days of culture (Fig. 1(c)). Cell number increased linearly with time for all substrates. The cell growth kinetics did not differ significantly between patterned substrates and the control after 3 days. For all the other time points measured, the patterned substrates had significantly higher (*p < 0.05, **p < 0.01) cell proliferation than the control. There was no significant difference between the patterned groups over 21 days. Proliferation rate was significantly higher (**p < 0.01) on TCP (up to 14 days) compared to non-patterned PDMS substrate.

It was noticable that keratocytes cultured on line patterned substrates were significantly (*p < 0.05) smaller than those on the control (non-patterned PDMS and TCP), lattice and pit patterned substrate (Fig. 1(d)). There was no significant difference between the size of cells cultivated on control and lattice patterned substrate.

### Cytoskeletal organization and chromatin condensation

**Figure 2:**
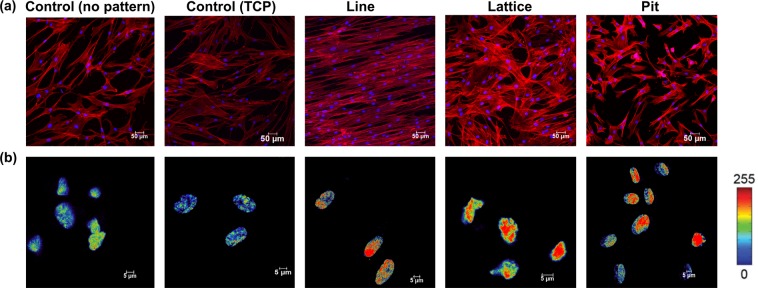
(**a**) Representative images of cells grown for 7 days on different PDMS patterned substrates and TCP. Cells were stained for f-actin (red) and nuclei using DAPI (blue). (**b**) Geometric constraint leads to chromatin condensation. Successive changes of the level of chromatin condensation as a result of cell growth on different PDMS micropatterned substrates and TCP. Intensities of DNA staining were digitized in 256 bits and color code. Highly condensed domains show higher fluorescence intensity with respect to the less condensed ones.

After staining for actin filaments, it could be seen that the keratocytes aligned in the direction of the microchannels on the patterned substrates while on the control substrates the cells orientation was random (Fig. 2(a)). On lattice and micropit patterned substrates, the cell orientation was less clear compared to line patterned substrates after 7 days of culture, but it was still noticeable that cells orientated themselves in the direction of the channels.

DAPI staining was used to investigate effect of topographical cues on size and shape of the cells nuclei^26,27^. Fluorescence intensity increased in proportion to chromatin condensation level. Nuclear reshaping is associated with more intense levels of chromatin condensation. Patterned substrate topographies resulted in higher chromatin condensation and nuclear reshaping when compared to the control (non-patterned PDMS and TCP) (Fig. 2(b)). The topographical cues also resulted in a reduced nuclear area.

### Cell orientation

**Figure 3:**
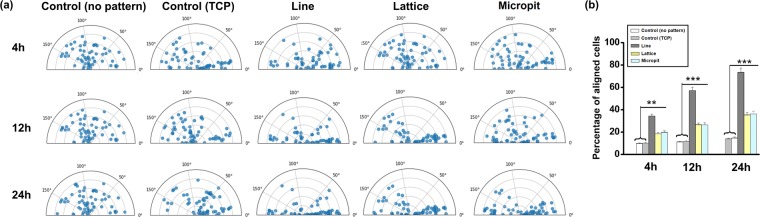
(**a**) Substrate topography induces orientation of the cell nucleus. Angular graphs show the different orientations experienced by nuclei (n = 60) in response to cell shape changes for various micropatterned substrate topographies. (**b**) Histograms of the cell orientation at different cultured time with respect to the pattern direction index of cells cultivated on different micropatterned substrates (***p < 0.001; **p < 0.01). Cells were considered aligned if the angle between the long axis and the grating was <10°.

At 4, 12, and 24 h following cell seeding, the orientation of the cells (nucleus) major axis was measured with respect to the pattern orientation on the substrate (Fig. 3). The cells on the patterned substrates significantly changed their nuclear and actin orientation, compared to the plane surface (Supplementary Fig. 1). Cells on the control substrates lacked any specific orientation. 24 h after seeding, cells on line (~73.5%) patterned substrates had more readily oriented themselves along the pattern, compared to the lattice (~35.2%), micropit (~36.21%) patterns and control (PDMS~13.8%, TCP~14.7%,) substrates. Nuclei orientation corresponded to the substrate topography’s orientation.

### Focal adhesions

**Figure 4:**
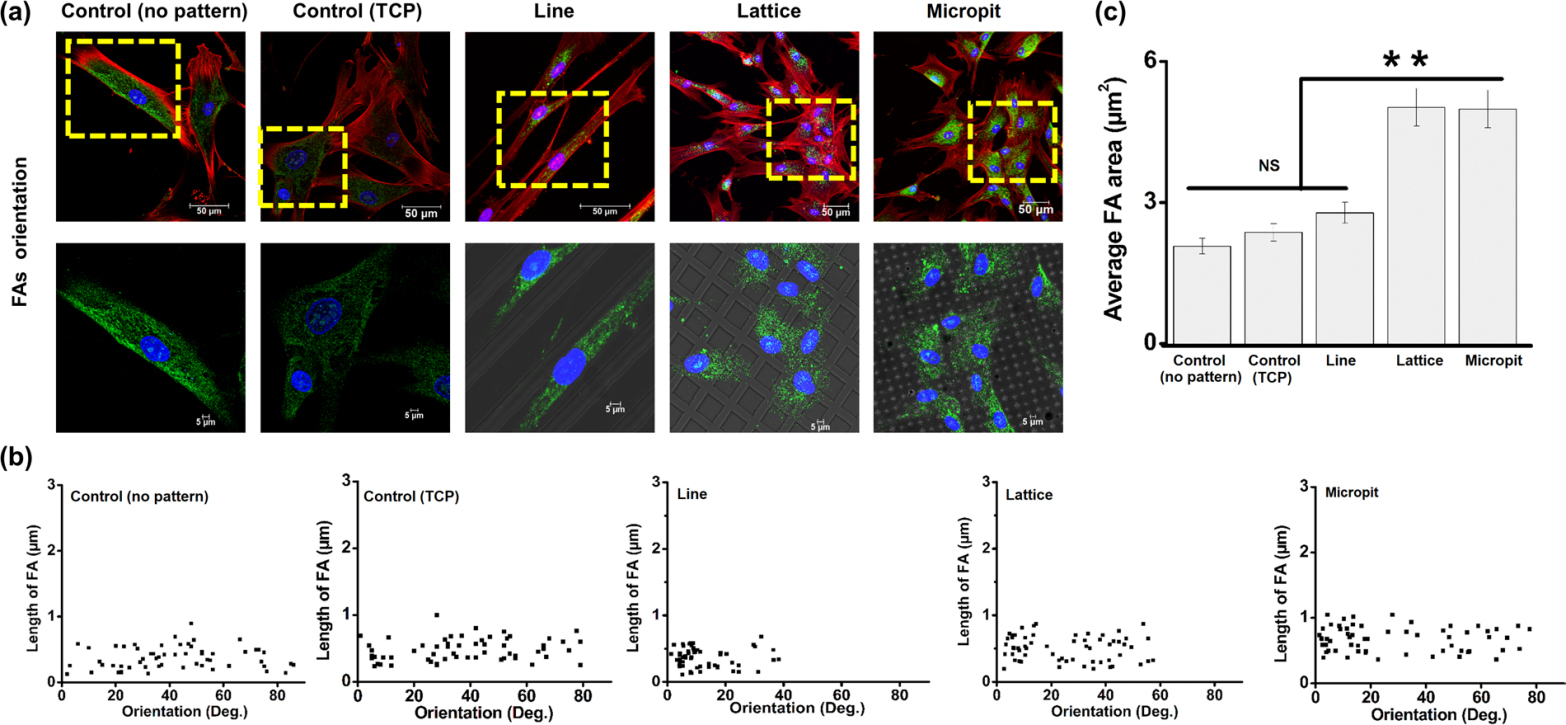
(**a**) FA orientation on different micropatterned substrate after 12 h of culture. Cells were stained for f-actin (red), vinculin (green) and nuclei (blue). FAs on patterned substrates were along the pattern direction. (**b**) Distribution of FA lengths with respect to pattern orientation. Length of FA (n = 60) measured on patterned substrates. For flat substrates (non-patterned PDMS and TCP), the calculation was performed with respect to the horizontal axis. (**c**) Average FAs area of different patterned substrate (**p < 0.01).

Vinculin staining was used to evaluate the cells focal adhesions (FAs) to the substrates. There were clear differences in the FAs between cell cultures on the patterned and the plane substrates (Fig. 4(a)). 12 h after seeding, FAs appeared diffuse within the cells’ cytoplasm on all samples. On the control substrates, FAs were bright and well defined but lacked any defined orientation. FAs stained more intensely on the line patterned substrates and were also observed at the terminal points of actin fibers on the substrates. FAs were present on top of the ridges between microchannels and were directed along the line orientation. The few FAs that were not directed along the pattern direction still appeared to be aligned and connected via single actin filaments on the surfaces that had micropatterns. Cells on lattice and micropit patterned substrates had less oriented FAs along the pattern direction compared to line patterned substrates. While FA length was similar across all substrates (Fig. 4(b)), focal adhesion area (N = 60) was significantly higher (**p < 0.01) on lattice patterned (5.023 ± 0.35 μm^2^) and micropit patterned (4.987 ± 0.39 μm^2^) substrates compared to line patterned (2.782 ± 0.2 μm^2^) and control (PDMS 2.072 ± 0.17 μm^2^, TCP 2.365 ± 0.19 μm^2^) substrates (Fig. 4(c)).

### Characteristics of filopodia

**Figure 5:**
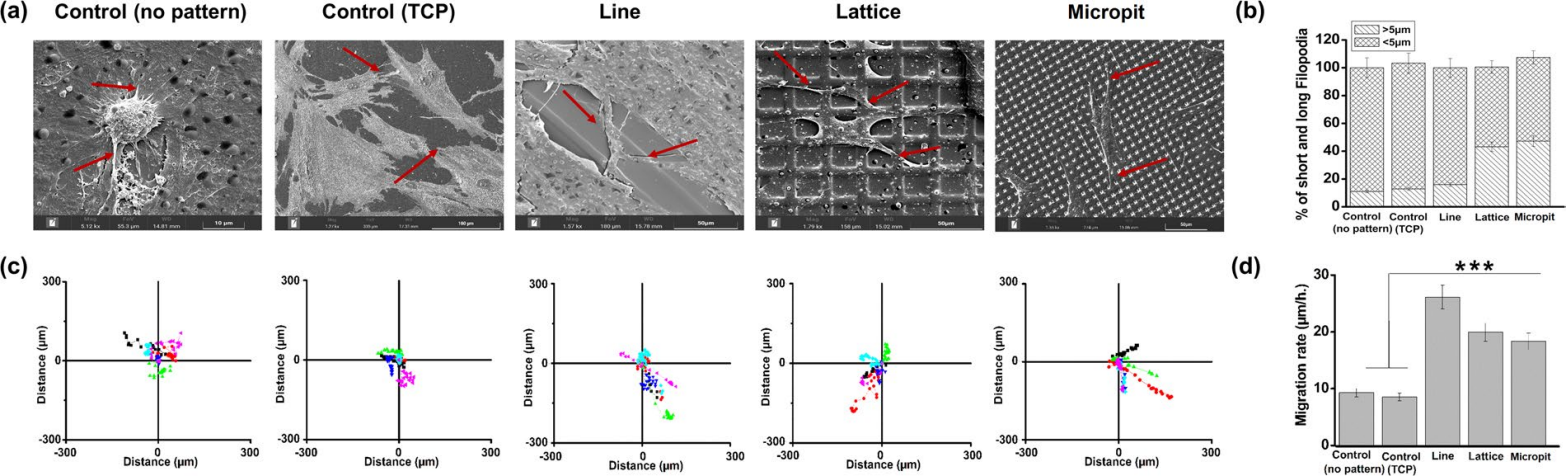
(**a**) Representative SEM images of filopodia on flat (non-patterned PDMS and TCP) and different micropatterned PDMS substrates. Arrows indicate filopodia. (**b**) Stacked histograms reporting the percentage of short (less than 5 μm) and long (more than 5 μm) filopodia on flat and different micropatterned PDMS substrates. (**c**) Migration rates of keratocytes cultured on the on flat and different micropatterned PDMS substrates. Trajectories of six cells cultured on each substrates imaged at 15 min intervals for 6 h. (**d**) Mean migration rates of cells cultured on each surface (n = 6 cells per surface, ***p < 0.001).

Filopodial probing, establishment of FAs and their growth are the precursors to cell spreading and elongation. On the control substrates (PDMS non-patterned), cells had a round morphology and the filopodia were straight. On TCP, cells are flat with short filopodia. On all patterned substrates, cells have a flatter, more spread morphology and they displayed some bent filopodia. The bending follows the pattern direction (Fig. 5(a)). Average filopodia length was significantly higher (*p < 0.05) on lattice and pit patterned substrate compared to control (PDMS non-patterned and TCP) and line patterned substrate (Fig. 5(b)). The average length did not exceed 8.25 ± 1.2 μm while most of the filopodia were under 5 μm in length.

### Direction of cell migration

Since microchannels affected FA maturation, spatial distribution, cell orientation and elongation, it would be logical to assume that the surface topography would affect cell migration. Keratocyte migration rates were analyzed using time-lapse images of the cells on the surfaces. The starting point of each cell was mapped onto the origin (0, 0) of the coordinate system used for plotting the cell positions (Fig. 5(c)). Cell migration trajectories on the control substrates were evenly distributed. For the patterned substrates, there was a correlation between cell trajectory and pattern orientation. This co-alignment was most conspicuous for the cells on the line pattern. Mean cellular migration rate was significantly higher on the patterned substrates, than on the plane substrate (p < 0.001, Fig. 5(d)). Among the patterned substrates, the line patterned substrates mean cellular migration rate (~26.14 μm/h) was not significantly different from that of the lattice (~19.94 μm/h) and micropit (~18.32 μm/h) patterned substrates.

### Characterization of ECM

**Figure 6:**
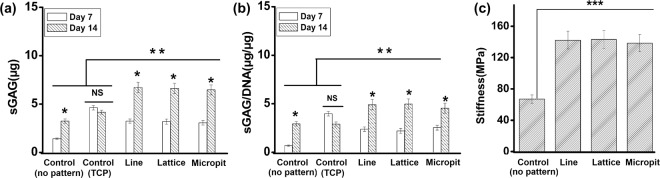
(**a**) GAG released by cells as quantified by DMMB assay and (**b**) GAG normalized to DNA after 7 and 14 days in culture. (**c**) ECM stiffness on different substrates recorded after 7 days in cultured, quantified by force-displacement curve method using AFM (***p < 0.001; **p < 0.01; *p < 0.05).

sGAG synthesis from the cells cultured on the different substrates (Fig. 6(a)) was significantly higher for the patterned substrates, compared to the controls at day 14 (line = 6.62 μg; lattice = 6.62 μg; micropit = 6.47 μg, controls = 3.24 μg (PDMS) and 4.16 μg (TCP)). When sGAG was normalized against DNA present at days 7 and 14, the trend remained similar (Fig. 6(b)). After 7 days of culture, it was noted that the extracellular matrix deposition had stiffened the substrates (Fig. 6(c)) and this was significantly more for the patterned substrates than for the plane substrates.

### Immunofluorescent staining and gene expression

**Figure 7:**
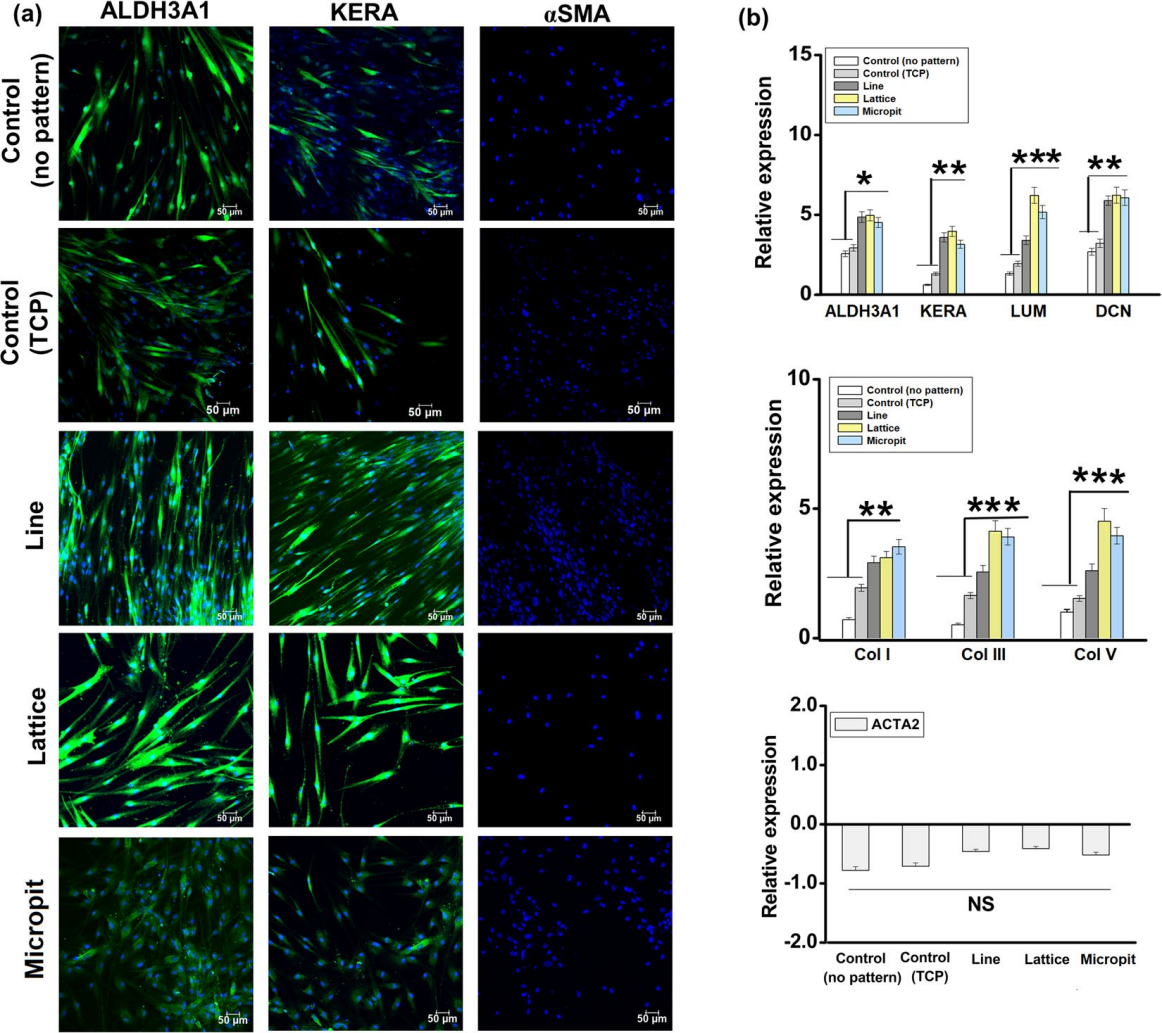
(**a**) Immunofluorescent staining of keratocytes specific proteins ALDH3A1 and keratocan and myofibroblastic marker αSMA (all green) and nuclei (blue) after 14 days in culture. (**b**) Fold change gene expression of keratocyte specific markers ALDH3A1, keratocan (KERA), lumican (LUM) and decorin (DCN), collagen ECM markers COLI, COLIII and COLV and myofibroblasts marker α-smooth muscle actin (ACTA2) as quantified by RT-PCR (***p < 0.001, **p < 0.01 and *p < 0.05).

Immunofluorescent staining was used to analyze markers related to keratocyte and myofibroblastic phenotypes (Fig. 7(a)). All the substrates had cells with the keratocyte markers ALDH3A1 and keratocan. None of the substrates showed any detectable levels of αSMA, a myofibrotic marker.

After 21 days of culture, RT-PCR was used to quantify gene expression. Cells on micropatterned substrates had increased expression of the markers ALDH3A1, keratocan, decorin and lumican, compared to the control substrates (Fig. 7(b)). Collagen types I, III, and V are the most common collagen components of corneal stroma and all three also had significantly higher levels of expression for the patterned substrates. Significantly higher (*p < 0.05) expression of lumican and collagen types III and V was recorded for lattice and micropit patterned substrates, compared with line patterned substrates. αSMA (ACTA2) expression was not influenced by the nature of pattern.

## Discussion

This study demonstrates that the behavior of keratocytes is influenced by the topography of their surrounding microenvironment. The linear pattern resulted in elongated and aligned cells while the lattice and micropit patterns resulted in more spread cells with less alignment. Despite these differences, many of the cells other characteristics were similar on both topographies compared to unpatterned substrates including increases in keratocyte specific gene expression, sGAG release and cell stiffness. While several studies have previously examined the effect of parallel microgrooves or nanogrooves on keratocytes^24–26,28^ this is the first time that orthogonal substrates have been used with this cell type. It is also pertinent to highlight that this study did not design the orthogonal lattice to mimic the collagen fibril structure in human stroma. Similarly, the channels on the linear patterned substrate do not replicate the stromal fibrils size. The goal was to use topographical cues to deliberately alter the keratocytes morphology and analyze any resulting change in the cell behavior.

Cell morphology and orientation were clearly influenced by the micropatterns used in this study. Previous works have shown that cell organelles like the centrosome, nucleus and golgi apparatus can orient themselves responding to external, physical stimuli^29–31^. Changes in morphology and cell size have also been shown to impact vital cellular processes such as growth, morphology, differentiation and death^32^. Interestingly in our study, despite differing cell morphologies on the three different patterned substrates, cells on each substrate proliferated at the same rate. Changes in the shape and size of the cell’s nucleus also affects its behavior and are linked to chromatin condensation. In our study, DAPI uptake levels were correlated to total DNA and its condensation level^30^. The ratio between integrated fluorescence intensity and nucleus volume was used to calculate an average spatial density that in turn provides a reliable indicator for average chromatin condensation^30,32^.

Cell proliferation was increased due to the presence of microchannels on the substrates. This might suggest activation of keratocytes since these cells are normally quiescent but can respond to injury by undergoing mitosis^4^. During wound healing, keratocytes trans-differentiate into a contractile myofibroblasts phenotype that is identifiable by the presence of αSMA. Keratocytes cultured *in vitro* in the presence of serum or TGF-β1 can also exhibit myofibroblastic phenotype^33–35^. In our study, keratocytes were initially cultured in serum supplemented medium but switched to serum free medium prior to seeding on the substrates. The lack of staining for αSMA within the cells and the lack of detectable changes in αSMA gene expression suggest that myofibroblastic differentiation was absent. Some actin stress fibers were present, although it was previously noted that keratocytes can retain a native keratocyte phenotype and exhibit stress fibers under serum free condition when insulin is used to supplement the media^36^.

Keratocytes can sense their environment through lamellipodial and filopodial extensions. Such probing can be used to contact other nearby cells or assist the cells to explore their surroundings. Keratocytes are mostly sedentary and through interconnections between cells, they form a cellular syncytium^37^. In this study, the microchannels appeared to interfere with cell filopodial probing and the establishment and growth of focal adhesion. The subsequent extension of the adhesions may have been obstructed by the topographic patterns^38^. Filopodia should be long enough to reach the valleys formed by the channels and therefore should not preclude the formation of new focal adhesions, however focal adhesions were limited to the top of the ridges on the substrates in this study. As observed by Albuschies & Vogel (2013), for high angles between filopodia and the adhesion surface (exceeding 12° on glass), focal adhesion formation is inhibited^39^. This could be due to the higher than normal stress on a filopodial extension when the angle increases, thus disrupting the integrin-ligand complex. It is conceivable that filopodia trying to reach the bottom of channels would be subjected to similar high angles with respect to the adhesion surface, hence adhesions only formed on the ridge tops. Focal adhesion area was also influenced by the surface topography. In turn, adhesion area strongly modulates adhesion strength and integrin binding^40^ and may act as a vital contributor for increased length of filopodia^41^.

The most common, negatively charged macromolecules found in the corneal-stromal ECM are GAGs. The release of GAGs by cells was increase on micropatterned substrates when compared to non-patterned substrates although there was no difference between the three patterns. These molecules contribute towards corneal transparency, nerve growth and cell adhesion^42^. GAGs like chondroitin sulfate and keratan sulfate have a necessary role in controlling inter-fibril distances and transparency of the cornea^43^. The increase production of extracellular matrix components such as GAG is important for when trying to regenerate corneal stroma either by replacing a scaffold over time or by using a self-assembly approach, where the cells need to produce sufficient ECM in culture to form a functional tissue^44^. Micro-patterns on a substrate surface and substrate stiffness can induce cues to improve formation and alignment of multinucleated myotubes, similar to myofibers,that can exhibit spontaneous contractions along the pattern’s longitudinal direction^45^.

Increased expression of the keratocyte specific genes ALDH3A1-a prominent crystalline protein, keratocan, lumican and decorin by cells on substrates with the microchannels supports the idea that topographical cues are important for maintaining the native keratocytes phenotype. In addition, micropatterned substrates enhanced expression of collagens found in the stromal matrix. Then *et al*. (2011), have also previously shown that specific genes expressed by keratocytes could be modulated by culturing the cells on microchannels^46^, although the genes examined were different to those examined in our work. Zhang *et al*. (2017), examined the effect of nanochannels on the expression of lumican, keratocan, collagen I and collagen V and while there were some differences to cells cultured on flat substrates, these were not found to be statistically significant^25^. In our study, keratocytes cultured on microchannels did up-regulate these specific genes. This variation in results could be due to several reasons including differences in the size of channels and different material being used. It has previously been shown that the size and geometry of microchannels or nanochannels can affect the gene expression of other cell types^47^ while factors such as material stiffness are known to influence the behavior of keratocytes^48–51^. In our study, there was a significant difference in the expression of only three genes (lumican, collagen III and collagen V) between cells on the aligned and orthogonal substrates and there were no differences in gene expression between cells on the lattice and micropit substrates. These results indicate that while topographical patterns had a significant effect on the cells gene expression, many genes associated with a native keratocyte phenotype, such as keratocan and ALDH3A1, remain unchanged on the different types of pattern examined despite differences in cell morphology. This suggests that cell morphology or gene expression when examined in isolation may be insufficient to appreciate differences in the cells phenotype and therefore characterization of both is required.

In conclusion, it has been shown that while topographical cues can be used to influence keratocyte behavior, several different factors including morphology, focal adhesions, gene expression and matrix deposition should be examined to gain a clearer insight on how the topography affects the cells. From our data, it appears that orthogonal patterned substrates appear to be the best at promoting a native keratocyte phenotype. This information could be useful for developing substrates assist in the restoration of a keratocyte phenotype after expansion in serum supplemented media and it could be applied to the design of scaffolds for corneal stromal regeneration, although further study would be required to determine if this these 2D findings could be applied in 3D.

## Materials and methods

### Fabrication of patterned (line and lattice) polydimethylsiloxane (PDMS) substrate

Micro patterned silicon master molds were fabricated using facilities at Centre for Research on Adaptive Nanostructures and Nanodevices (CRANN, Class 1,000 Clean room, Trinity College Dublin). Protocol followed has been described earlier^52^. Briefly, molds were fabricated by standard soft lithography (Microcontact printing) Laser Mask Writer (Heidelberg DWL66), reactive ion etching, and low-pressure chemical vapor deposition for silicon oxide coating. There were three master molds, each 1 × 1 cm in dimension: (a) 5 μm channels, 20 μm space, aligned (line structure) and (b) 5 μm channels, 20 μm space, crossed (lattice structure) (c) 4 μm channels, 6.5 μm space (micropit structure). The PDMS patterned substrates (1 cm × 1 cm) were characterized by scanning electron micrography (SEM, SUPRA 35 VP,Carl Zeiss) and white light interferometry (Omniscan MicroXam-non-contact optical method) to measure the surface roughness (RMS: root mean square) and the depth of microgrooves channels.

### Cell culture

Cells were isolated and expanded from human corneas as previously described^23^ in accordance with the Declaration of Helsinki. The written informed consent obtained from donor or next of kin for the collecting tissue samples. Ethical approval for the use of human tissue in this study was provided by the Trinity College Dublin, University of Dublin, School of Medicine Research Ethics Committee. The cell culture media for the human keratocytes (passage 3) comprised of Dulbecco’s Low Glucose Modified Eagles Medium (DMEM) (Hyclone; Thermo Fisher Scientific) supplemented with 10% fetal bovine serum (FBS, Gibco) and 1% penicillin and streptomycin solution (Pen/Strep, Invitrogen). The patterned substrates (1 × 1 cm^2^) were sterilized by UV exposure (30 min) and soaked in low glucose DMEM medium (without FBS and pen/strep) for 4 hours. Two hours prior to cell seeding, the matrices were partially dried. This ensured better cell penetration. Ten microliters of cell suspension in media, containing about 10^3^ cells, were seeded dropwise onto each matrix. For the one hour following cell seeding, the matrices were maintained in a humidified environment, at 37 °C and 5% CO_2_, to enhance cell adhesion. Cell seeded substrates were maintained in DMEM/F12 (Hyclone; Thermo Fisher Scientific) medium. This medium was supplemented with L-ascorbic acid, Insulin-Transferrin-Selenium (Gibco), and penicillin/streptomycin. Media was replaced on every alternate day. The PDMS substrates were not coated with any protein that could enhance cell adhesion, e.g., fibronectin, to better understand the impact of substrate topography on cell behavior. Substrates were cultured in serum free media to maintain better keratocyte phenotype.

### Cellular proliferation and cytoskeletal organization

Cell proliferation was assessed at multiple day-points over the 21 day culture period using PrestoBlue reagent (Molecular Probes; Invitrogen, Carlsbad, CA), while using the manufacturer’s protocol. To observe cytoskeletal organization, following 7 days of culture, cell laden substrates were stained with Phalloidin-TRITC (actin filaments) and 4′,6 -diamidino-2-phenylindole (DAPI) (nuclei) respectively following manufacturer’s protocols. Leica SP8 confocal microscope was used for examining the cytoskeletal organization on the matrices with LAS X Advanced software being used for image post-processing.

### Analysis cells spreading area and degree of orientation

The visualization yielded by F-actin and DAPI staining was used to outline the cell perimeter and to calculate the cell areas^37^. Analyze Particles, from Fiji was used to obtain these areas. At each time point, for each substrate, 30 cells were analyzed.

TRITC-phalloidin stained cells, following 4, 12, and 24 hours of cell seeding, were analyzed using Moment Macro J v. 1.3 script in Fiji to obtain principal moments of inertia for the cells (assuming elliptical profiles). Cell orientation was defined as the angle (θ) between the principal axis of inertia of the elliptical form and a reference axis. θ varied between 0 and 180°. For reference axis, the pattern direction was used in the micro-grooved surfaces while the horizontal axis was used for the control surfaces (no grooves). When θ exceeded 10°, cells were considered to have random orientation and were otherwise considered to be aligned.

### Focal adhesions (FAs) and morphometric analysis

For focal adhesion analysis, 12 hours after cell seeding, Mouse monoclonal anti-vinculin antibody (Abcam) and Alexafluor-conjugated rabbit anti-mouse IgG (Abcam) was used to stain cell-laden constructs following manufacturer’s protocols. Actin and nucleus were counter stained as described previously. Substrates were then analyzed by confocal microscopy: Leica SP8 for imaging and LAS X Advanced for image post-processing. The methodology from Maruoka *et al*. (2012), was used for FA morphometric analysis (length vs orientation)^53^. Area of FA (n = 60) on different substrate was also calculated. ImageJ command “Measure” was used to measure FA length and area. FA lengths (n = 60) were analyzed with respect to pattern orientation.

### Apparent chromatin condensation

DAPI staining (particularly conjugates to the double-stranded DNA) was used to visualize chromatin and to analyze the spatial chromatin organization. Nuclear images at different focal positions along the z axis, every 0.2 mm interval, were acquired using confocal scanning microscopy (Leica SP8) and stacked. Images were sharpened with ImageJ plugins (shading correction, dark image subtraction, Deconvolution Lab) to extract quantitative information from them. As the sum of the intensity of each pixel, the integrated fluorescence intensity was quantified. The average spatial density is the ratio of total fluorescence intensity to nuclear volume and correlates with average chromatin packing ratio^26^. It is indicative of chromatin condensation.

### Filopodia feature analysis through SEM

After 24 h of seeding, fixed (4% PFA, 15 min, room temperature) cell laden substrates were examined for analysis of filopodia features through scanning electron microscopy (SEM, Zeiss Sigma 300, operating voltage of 10 kV).

### Cell migration analysis

After 4 h of cell seeding, cell migration studies were started and carried on for 6 h. Each cell was tracked until cell division occurred. Hence, we restricted our cell migration study to 6 h. The study focused on early intervals following seeding since the cell density is low during this period, helping us avoid impact of cell-cell interactions. Six representative areas of each substrate were selected for acquiring bright field images. Cells were tracked based on their centroid. Imaging was carried out every 15 min. Time-lapse videos were analyzed with METAMORPH (v. 6.1, Molecular Devices, Sunnyvale, CA, USA) in order to extract cell trajectories. Mean migration rate was defined as the total movement of the call per unit time and directionality was assessed as movement across vs movement along pattern direction^41^. Mean migration was averaged for six cells on each type of substrate.

### Gene expression by real-time Real Time-PCR

Real-time reverse transcription PCR was used to quantify relative gene expression after 21 days of culture following protocol in literature^54^. Gene expressions were normalized using the ΔΔCt method, against GAPDH.The primers (Applied Biosystems, Biosciences, Dublin, Ireland) evaluated were: αSMA (Hs00426835_g1), ALDH3A1 (Hs00964880_m1), decorin (DCN;Hs00754870_s1), glyceraldehyde-3-phosphate dehydrogenase (GAPDH; Hs02758991_g1), keratocan (KERA;Hs00559942_m1), and lumican (LUM; Hs00929860_m1), collagen type III (COL3A1; Hs00943809_m1), collagen type I (COL1A1; Hs00164004_m1), collagen type V (COL5A1; Hs00609133_m1_m1).

### Evaluation of Extracellular Matrix (ECM) characterization and stiffness

ECM formation on the cell laden substrates and the culture media was quantified using Dimethylmethylene blue (DMMB) binding assay (Blyscan; Biocolor Ltd, Antrim, UK) after 14 and 28 days of culture period to determine levels of sGAG (acid sulfated glycosaminoglycans) following manufacturer’s protocol. The stiffness of ECM generated by cells on all PDMS substrates after 7 days of culture was evaluated by contact mode Atomic force microscopy (AFM-Park NX10) using force-Displacement curve (Park Systems XEI software). All PDMS substrates had the same initial stiffness before the addition of cells regardless of topography. Due to harder surface on TCP, the stiffness of the ECM generated on this substrate could not be measured without risk of damaging the instrument.

### Immunocytochemistry

Following 14 days of cell culture, cell-laden substrates were pre-processed for immunocytochemistry analysis. After permeabilization and blocking, substrates were incubated at 4 °C in anti-ALDH3A1 1:50 (ab76976; Abcam, Cambridge, United Kingdom), anti-keratocan 1:50 (sc-66941; Santa Cruz, Heidelberg, Germany), and anti-α-smooth muscle actin (αSMA) 1:50 (ab7817; Abcam). To detect Keratocan and ALDH3A1, Donkey anti-rabbit AlexaFluor 488 (ab150073; Abcam) was used. For αSMA, goat anti-mouse biotin was used, succeeded by Extr Avidin-FITC (B7151 and E2761). Nucleus was counter stained. Confocal microscopy was carried out using the Leica SP8 and post-processing used LAS X Advanced software.

### Statistical analysis

The R statistical platform was used for all statistical analysis. One-way ANOVA followed by Tukey’s significant difference test used to compare data. Significant differences have been marked as ***p < 0.001; **p < 0.01; *p < 0.05. Data presentation is in the form mean ± standard deviation (SD). Unless otherwise specify, sample size was 3.

## Supplementary information

Supplementary information.
