# Supplementary material for: Influence of micropatterned substrates on keratocyte phenotype
Source: Sci Rep. 2020 Apr 21;10:6679. doi: 10.1038/s41598-020-62640-5 (PMC7174307; doi:10.1038/s41598-020-62640-5)
Supplement: Supplementary file 1 — Supplementary information. [file 41598_2020_62640_MOESM1_ESM.pdf]

Influence of micropatterned substrates on keratocyte phenotype

Promita Bhattacharjee[1,2], Brenton. L. Cavanagh [3] and Mark Ahearne[1,2*]

[1]Trinity Centre for Biomedical Engineering, Trinity Biomedical Sciences Institute, Trinity College Dublin, University of Dublin, Dublin, Ireland.

[2]Department of Mechanical and Manufacturing Engineering, School of Engineering, Trinity College Dublin, University of Dublin, Dublin, Ireland.

[3]Cellular and Molecular Imaging Core, Royal College of Surgeons in Ireland, Dublin, Ireland

* Corresponding Author: Mark Ahearne; Tel.: +353 1 8962359; fax: +353 1 6795554; E-mail address: ahearnm@tcd.ie

Supplemental Figure 1

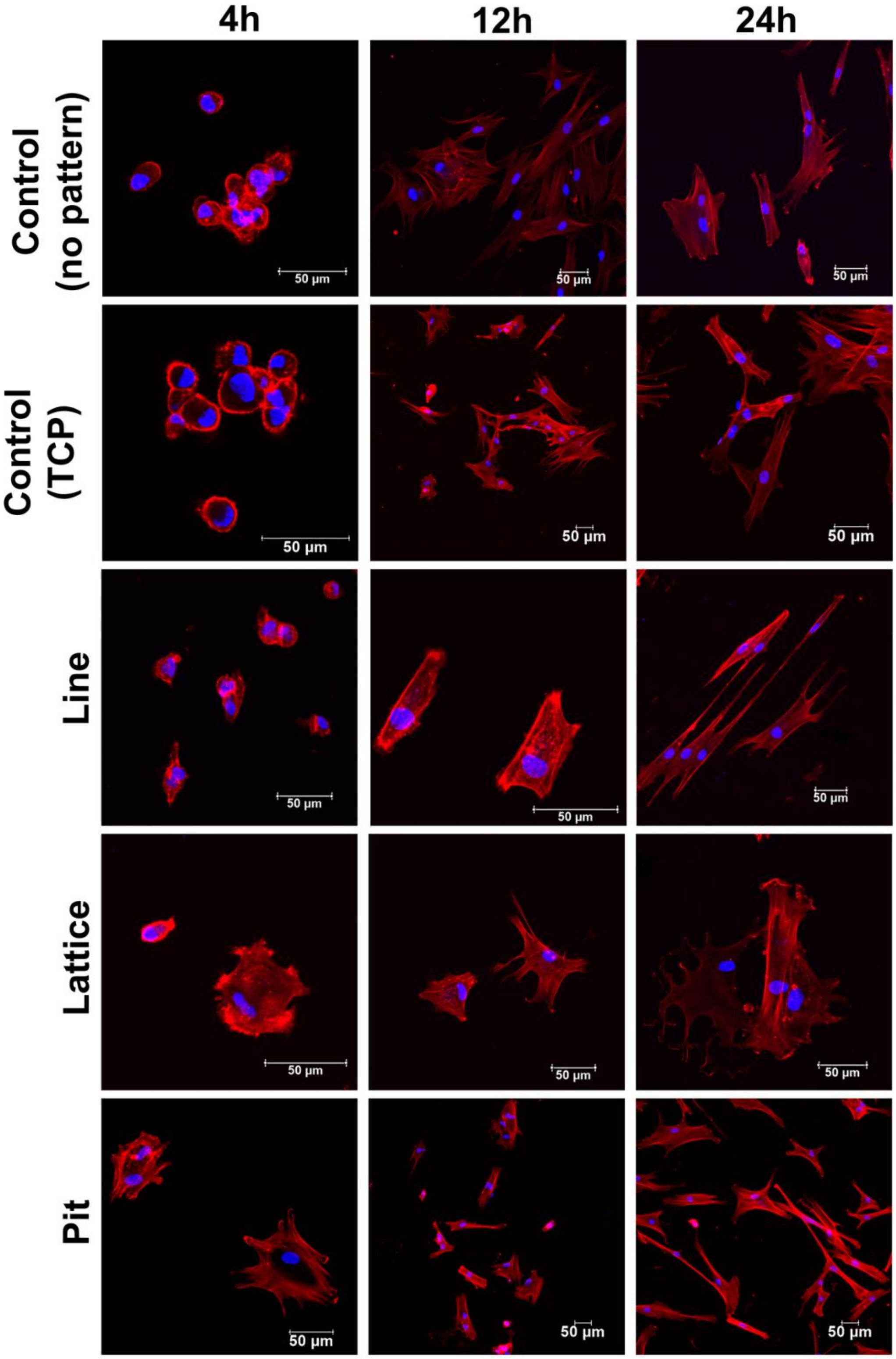

Supplement Figure 1: (a) Cell and nucleus orientation in respect of time on flat (non-patterned PDMS and TCP) and different micro patterned substrates. Cellular orientation was quantified using the confocal fluorescence images, actin (red) and nucleus (blue)
